# An acute eccentric exercise increases circulating myomesin 3 fragments

**DOI:** 10.1186/s12576-021-00789-y

**Published:** 2021-01-19

**Authors:** Minjung Lee, Jaehoon Shin, Tatsuya Kato, Kazue Kanda, Satoshi Oikawa, Jun Sakuma, Kaoru Sugama, Yasuo Kawakami, Katsuhiko Suzuki, Takayuki Akimoto

**Affiliations:** Faculty of Sport Sciences, Waseda University, 2-579-15 Mikajima, Tokorozawa, Saitama 359-1192 Japan

**Keywords:** Myomesin, Eccentric exercise, Muscle damage, Biomarkers

## Abstract

Discovery of blood biomarkers to evaluate exercise-induced muscle damage have attracted many researchers and coaches. This study aimed to determine changes in circulating myomesin 3 fragments as a novel biomarker for exercise-induced muscle damage. Nine healthy males performed 10 sets of 40 repetitions of one-leg calf-raise exercise by the load corresponding to the half of their body weight. Muscle symptoms were evaluated by a visual analog scale (VAS). Blood samples were collected before and 2, 4, 24, 48, 72, and 96 h post-exercise. Plasma myomesin 3 fragments levels were significantly increased at 96 h after the eccentric exercise. The myomesin 3 fragments levels were correlated with other biomarkers of muscle damage and the muscle symptoms. These results suggest that the circulating myomesin 3 fragments levels are potential biomarkers reflecting eccentric exercise-induced muscle damage.

## Introduction

Muscle damage is induced not only in pathological setting (i.e., muscular dystrophies) but also physiological setting (i.e., unaccustomed exercise and/or eccentric exercise), causing skeletal myofiber disruption, inflammatory cell infiltration, and muscle soreness [1–5]. There is a growing need for biomarkers in order to monitor disease progression and/or efficiency of treatments in patients with muscular dystrophies. Similarly, researchers in sport sciences have been looking for biomarkers to monitor muscle damage of athletes to plan/change their training since the exercise-induced muscle damage cause various muscle symptoms which can impair sport performance [6].

A number of biomarkers for muscle damage have been discovered so far. Among them, circulating intramuscular proteins, such as creatine kinase (CK) and myoglobin (Mb), are generally used in many clinical diagnoses [7, 8]. Delayed-onset muscle soreness (DOMS), changes in muscle tenderness, and the range of motion have also been used to indicate muscle symptoms of exercise-induced muscle damage [9, 10]. However, researchers continue to seek novel biomarkers for muscle damage to find more sensitive biomarkers that can precisely reflect magnitude of muscle damage and symptoms than the conventional ones [11].

Recent progress in proteomic technology offers a strategy for the comprehensive analysis of protein expression, which can be applied to the search for biomarkers. In fact, several proteomic studies, including ours, have already been reported to identify novel biomarkers for muscle damage in patients with muscle degenerative diseases or in athletes who engaged in eccentric exercise [12–15]. Rouillon et al. [14] revealed that two fragments of a myofibrillar structural protein, myomesin 3, were significantly increased in sera of patients with muscular dystrophies, who show progressive muscle disruption and regeneration, by using a comprehensive high-resolution mass spectrometry approach. Regarding exercise-induced muscle damage, they reported that the myomesin 3 fragments did not change after an acute bout of eccentric downhill running in mice [14]. However, it has not been determined whether the myomesin 3 fragments are responsible to exercise-induced muscle damage among human subjects.

In this study, we measured the levels of the circulating myomesin 3 fragments in healthy volunteers before and after 10 sets of 40 repetitions of eccentric one-leg calf-raise exercise. In our knowledge, this is the first study describing a significant increase of the myomesin 3 fragments in response to eccentric exercise.

## Methods

### Subjects

Nine untrained healthy males participated in the original investigation [16]. The mean (± SD) characteristics of the subjects were as follows: age 24.8 ± 1.3 years, body mass 62.3 ± 6.3 kg, and height 1.72 ± 0.05 m. The subjects had not been involved in any intense exercise or resistance training for at least two weeks before the exercise bout, and were not taking any drugs, nutritional supplements, or participating in recovery strategies, such as massage, stretching, or cryotherapy. The subjects were instructed to maintain their usual daily schedules during the experiment. The study protocol was approved by the Ethics Committee of Waseda University, Japan, in accordance with the Declaration of Helsinki [17], and the subjects gave their written informed consent.

### Experimental design

The subjects performed a calf-raise exercise, including repetitive eccentric muscle contractions, with their right leg on a force plate, as described previously [16]. Briefly, each subject rested on an exercise device specially designed for ankle plantar flexion, with the knee joint extended and the metatarsal bone resting on a stool. The slope of the backrest was 30°, so that the exercise load corresponded to approximately half of the subject’s weight (exercise load = body mass × sin 30°). With their right leg, subjects performed single-leg ankle plantar flexion exercise consisting of 10 sets of 40 repetitions with a 3-min rest between sets. The range of motion (ROM) of the ankle joint during the exercise was maintained between 20° (dorsiflexion position) and 15° (plantar flexion position) using an electronic goniometer (SG110/A, Biometrics, Newport, UK) with its ends attached to the distal-lateral part of the fibula and the lateral part of the foot. Each subject received visual feedback on his ankle joint ROM during the exercise via display of the joint ROM value on a personal computer. The exercise was performed in accordance with the rhythm of an electrical metronome at a speed of 60 counts/min; ankle dorsiflexion and plantar flexion were alternated and repeated every 1 s. All subjects completed a total of 400 repetitions of ankle plantar flexion. Delayed-onset muscle soreness (DOMS) was rated with a visual analog scale (VAS): a 100-mm line with “no pain” at one end and “extremely sore” at the other. The tenderness of the exercised muscle correlative to DOMS was assessed using the FP meter (SN-402, Navis, Japan) at 1 kg. The point of measurement was the middle point of medial gastrocnemius [16]. Blood samples were collected before and 2, 4, 24, 48, 72, and 96 h after exercise. Serum myoglobin (Mb), creatine kinase, (CK) and lactate dehydrogenase (LDH) were measured as described previously [16, 18]. Data for serum levels of Mb, CK, and LDH as well as muscle symptoms (ROM, VAS, and tenderness) were obtained from the previous studies [16, 18].

### Immunoblotting analysis

The collected plasma was mixed with complete protein-loading buffer containing 50 mM Tris–HCl (pH 6.8), 1% SDS, 10% glycerol, 20 mM dithiothreitol, 127 mM 2-mercaptoethanol, and 0.01% bromophenol blue, supplemented with protease inhibitors (Roche) and phosphatase inhibitors (Sigma-Aldrich, St. Louis, MO, USA). The plasma samples were transferred to microfuge tubes, heated for 1 min at 95 °C, and centrifuged in a microfuge for 5 min at 12,000×*g* at room temperature. The plasma samples were then loaded onto 7.5% gels (depending on the molecular weight of the protein) for SDS-polyacrylamide gel electrophoresis (PAGE), transferred to a nitrocellulose membrane, and immunodetected with an enhanced chemiluminescence kit (ECL prime, Amersham) using the LAS-3000 Imaging System (Fuji Film, Tokyo, Japan), as described previously [19]. Antibodies directed against the myomesin 3 fragments were used for the immunoblotting analysis: anti-myomesin 3 (MYOM3) polyclonal antibody (17692–1-AP; Proteintech, Chicago, IL, USA). The secondary antibodies used were horseradish peroxidase (HRP)-conjugated goat anti-rabbit IgG antibody (Amersham). The proteins were quantified with the ImageJ software (NIH, Bethesda, MD, USA).

### Statistical analysis

The data were analyzed with Friedman test. When significant time effects were evident, pairwise comparisons were analyzed by the Dunn’s test with Bonferroni’s correction. Associations between data were analyzed with Pearson’s correlation coefficient (r). Statistical significance was set at *p* < 0.05, and the data are presented as means ± standard error (SE). All statistical analyses were performed by SPSS statistics ver. 26 (IBM, USA).

## Results

### Changes in circulating myomesin 3 fragments after an acute eccentric exercise

**Fig. 1:**
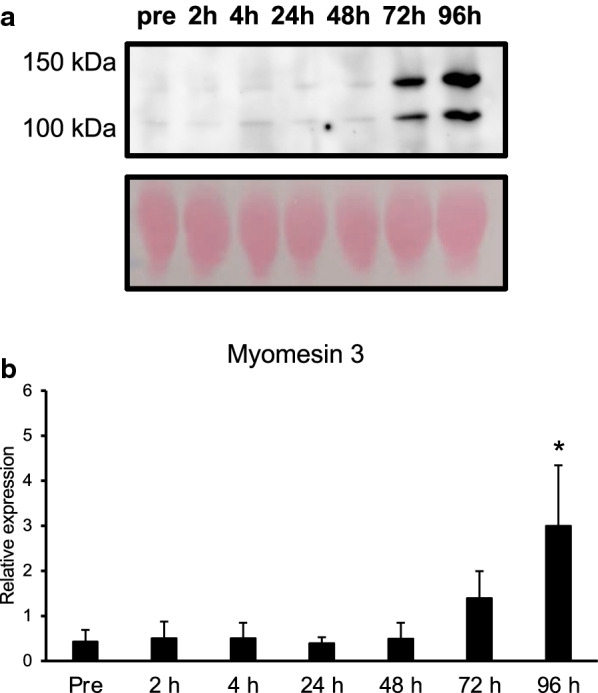
Changes in circulating myomesin 3 fragments in response to eccentric exercise. **a** Representative images of western blot for myomesin 3 and Ponceau S staining. **b** Relative expression of plasma myomesin 3 fragments levels of pre, 2, 4, 24, 48, 72, and 96 h after an acute bout of eccentric exercise. Values are expressed as means ± SE (*n* = 9). *p < 0.05 vs Pre

We measured the levels of the myomesin 3 fragments in plasma of nine subjects before and after an acute eccentric exercise. The myomesin 3 fragments were detected at 100 kDa and 130 kDa (Fig. 1a), respectively, as previously described [14]. We found that the myomesin 3 fragments increased significantly 96 h after the exercise (Fig. 1b, *p* < 0.05), although the changes in the circulating myomesin 3 levels varied among subjects (Additional file 1: Fig. S1).

### Relationships between the myomesin 3 fragments levels and other blood markers of muscle damage

**Fig. 2:**
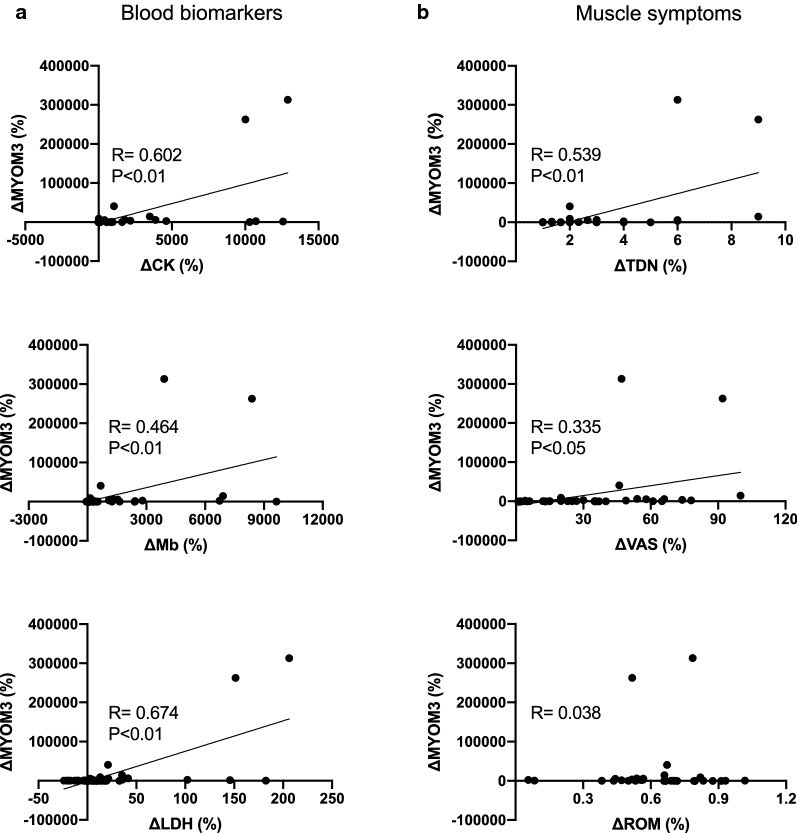
Relationships between myomesin 3 fragments levels and circulating biomarkers of muscle damage, and muscle symptoms after eccentric exercise. **a** Relationships between Myomesin 3 (MYOM3) vs Creatine kinase (CK), lactate dehydrogenase (LDH), and myoglobin (Mb). **b** Relationships between Myomesin 3 (MYOM3) vs ankle range of motion (ROM) in dorsal flexion, Muscle tenderness (TDN), and visual analog scale (VAS) for delayed-onset muscle soreness (DOMS). All data are percent changes (Δ) between pre-exercise and post-exercise values. *p < 0.05, **p < 0.01

As shown in Table 1 and Fig. 2a, there were positive correlations between the percent changes in the myomesin 3 fragments levels and that in CK (*R* = 0.602, *p* < 0.01, See also Additional file 2: Table S1), Mb (*R* = 0.464, *p* < 0.01, See also Additional file 2: Table S2), and LDH (*R* = 0.674, *p* < 0.01, See also Additional file 2: Table S3) levels (Table1, Fig. 2a).

**Table 1:** Pearson’s correlation coefficient matrix of myomesin 3 and muscle damage markers

	MYOM3	CK	Mb	LDH
MYOM3		0.602**	0.464**	0.674**
CK			0.810**	0.944**
Mb				0.793**
LDH				

Myomesin 3 (MYOM3), creatine kinase (CK), myoglobin (Mb), lactate dehydrogenase (LDH). All data are calculated as changes for the pre-exercise values. **p < 0.01

### Relationships between the myomesin 3 fragments levels and muscle symptoms

We next determined whether the changes in the circulating myomesin 3 levels associated with muscle symptoms, such as muscle tenderness (TDN), DOMS, and ROM. As shown in Table 2 and Fig. 2b, we observed positive correlations between percent changes in myomesin 3 levels and that in TDN (*R* = 0.539, *p* < 0.01, See also Additional file 2: Table S4) and VAS (visual analog scale for DOMS, *R* = 0.335, *p* < 0.05, See also Additional file 2: Table S5). Changes in ROM (*R* = 0.038, *p* = 0.838) was not correlated with myomesin 3 levels (Table 2, Fig. 2b, See also Additional file 2: Table S6).

**Table 2:** Pearson’s correlation coefficient matrix of myomesin 3 and muscle symptoms

	MYOM3	TDN	VAS	ROM
MYOM3		0.539**	0.335*	0.038
TDN			0.813**	-0.127
VAS				-0.451**
ROM				

Myomesin 3 (MYOM3), muscle tenderness (TDN), and visual analog scale (VAS) for delayed-onset muscle soreness (DOMS), and ankle range of motion (ROM) in dorsal flexion. All data are calculated as changes for the pre-exercise values. *p < 0.05, **p < 0.01

## Discussion

The major finding of this study is that circulating myomesin 3 fragments increased in response to an acute bout of eccentric exercise. To our knowledge, this is the first report describing an increase of the myomesin 3 fragments after eccentric exercise in humans.

There are three isoforms of myomesin: myomesin 1, myomesin 2 (M-protein), and myomesin 3. These myomesins are the principal components of the cytoskeletal structure called the M-band that cross-links anti-parallelly arranged myosin filaments and titin filaments in the middle of the sarcomere [20, 21]. It has been suggested that myomesin is a molecular spring whose elasticity guards the stability of the sarcomere similar to titin [22]. Expression of myomesin 3 was found mainly in type IIa fibers of skeletal muscle, while type IId/x fibers express more myomesin 2 and myomesin 1 is expressed in all muscle fibers [22]. Since type II muscle fibers are more susceptible to eccentric exercise-induced muscle damage [23], it seems to be reasonable that type II fibers were selectively disrupted and the myomesin 3 fragments were released into blood stream in response to eccentric exercise.

Rouillon et al. [14] revealed that two fragments of myomesin 3 are detected in sera of patients with muscular dystrophies as well as model mice for muscular dystrophy (mdx mouse), while myomesin 3 fragments are barely detected in sera of healthy individuals and wild-type animals in their original investigation. Then, Rouillon et al. [14] further tested if an acute bout of eccentric exercise alters circulating levels of the myomesin 3 fragments in mice. They found that the levels of the myomesin 3 fragments did not change after an acute eccentric downhill running exercise in mice. In contrast, we found that an acute eccentric calf-raise exercise increased the myomesin 3 fragments in humans. To explain the different behavior of the myomesin 3 fragments in mice and humans, we suggest that the degree of muscle damage was different among these studies. It is possible that the eccentric downhill running exercise was not sufficient to induce muscle damage in mice because the serum CK levels showed no change in mice [14], while we found a significant increase in CK after the eccentric calf-raise exercise in the present study.

Rouillon et al. also reported that circulating levels of myomesin 3 showed lower inter-individual variances than those of CK among patients with muscular dystrophies [14]. On the other hand, we observed that inter-individual variances still existed in the myomesin 3 levels similar to the other biomarkers for muscle damage in this study. Muscular dystrophy patients have identical genetic factors that might cause less diversity of muscle damage, while healthy subjects may have less in common than muscular dystrophy patients and have more various factors influencing exercise-induced muscle damage. Firstly, genetic polymorphisms in sarcomeric proteins and growth factors might vary the degree of exercise-induced muscle damage among the healthy subjects [24, 25]. Also, considering that fast-twitch fibers show severer eccentric contraction-induced muscle damage than slow-twitch fibers [23], distinct composition of fast-twitch muscle fiber in each participant might have influence on the degree of exercise-induced muscle damage.

There was no clear difference in the time course of cytosolic CK, LDH, Mb, and structural myomesin 3 appearance in the circulation after the eccentric exercise, despite the different roles and distributions in muscle cells. The reason for the similar kinetics of these biomarkers in the circulation is that the mechanisms via which each muscle damage marker leaks into the blood may be the same [26]. Generally, damage to the sarcolemma causes intracellular proteins to leak into the blood, which is a major cause of the increase in cytosolic proteins in the blood following eccentric exercise [26].

The present study has limitations. Various factors, such as gender, age, muscle mass, genetic factors, selected muscle groups, training frequency, and frequency of daily exposure to eccentric contractions, cause differences in exercise-induced muscle symptoms among individuals [26, 27]. We could not avoid the subjects being exposed to eccentric contractions on a daily basis although the subjects in this study performed limited resistance training during the experimental period.

In conclusion, an increase in the plasma myomesin 3 fragments is potentially a biomarker of exercise-induced muscle damage because it reflects the changes that occur in other markers of muscle damage and in the muscle symptoms observed after eccentric exercise.

### Supplementary Information

**Additional file 1: Figure S1.** Entire images of Western blot for fragmented myomesin 3. Entire images of Western blot for myomesin 3 and Ponceau S staining. Pre, 2, 4, 24, 48, 72, and 96 h after an acute bout of eccentric exercise.

**Additional file 2: Table S1.** Pearson’s correlation coefficient matrix of myomesin 3 and creatinine kinase at each time point. **Table S2.** Pearson’s correlation coefficient matrix of myomesin 3 and myoglobin at each time point. **Table S3.** Pearson’s correlation coefficient matrix of myomesin 3 and lactate dehydrogenase at each time point. **Table S4.** Pearson’s correlation coefficient matrix of myomesin 3 and muscle tenderness at each time point. **Table S5.** Pearson’s correlation coefficient matrix of myomesin 3 and DOMS at each time point. **Table S6.** Pearson’s correlation coefficient matrix of myomesin 3 and ROM at each time point.

## Data Availability

The datasets generated and/or analyzed during the current study are included in this study and previous studies [16, 18].
