# Supplementary figures and images for: An acute eccentric exercise increases circulating myomesin 3 fragments
Source: J Physiol Sci. 2021 Jan 19;71:4. doi: 10.1186/s12576-021-00789-y (PMC10717673; doi:10.1186/s12576-021-00789-y)

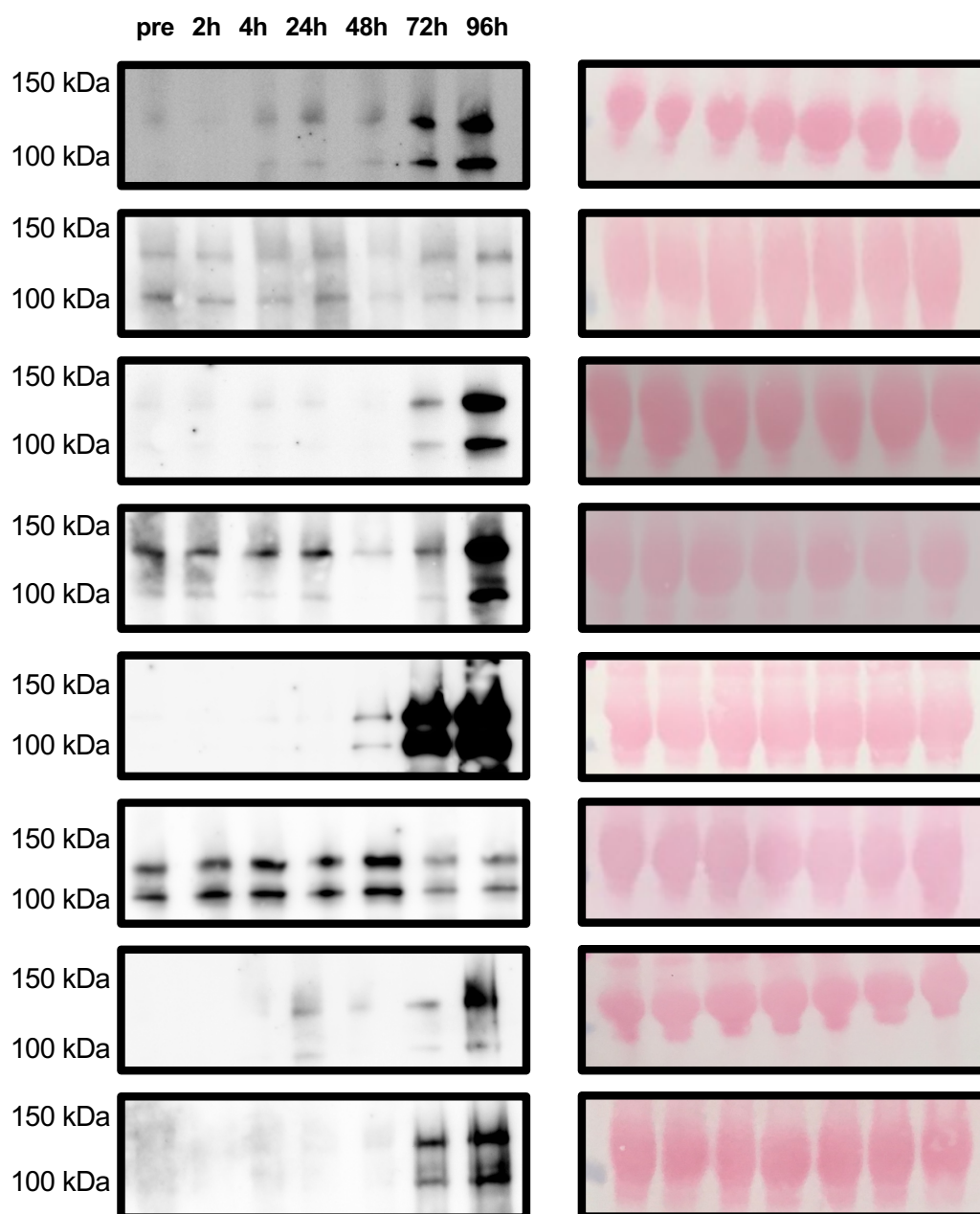

**Supplementary figure 1.**

Supplement: Supplementary file 1 — Additional file 1: Figure S1. Entire images of Western blot for fragmented myomesin 3. Entire images of Western blot for myomesin 3 and Ponceau S staining. Pre, 2, 4, 24, 48, 72, and 96 h after an acute bout of eccentric exercise. [file 12576_2021_789_MOESM1_ESM.pdf]
