# Supplementary material for: An acute eccentric exercise increases circulating myomesin 3 fragments
Source: J Physiol Sci. 2021 Jan 19;71:4. doi: 10.1186/s12576-021-00789-y (PMC10717673; doi:10.1186/s12576-021-00789-y)
Supplement: Supplementary file 2 — Additional file 2: Table S1. Pearson’s correlation coefficient matrix of myomesin 3 and creatinine kinase at each time point. Table S2. Pearson’s correlation coefficient matrix of myomesin 3 and myoglobin at each time point. Table S3. Pearson’s correlation coefficient matrix of myomesin 3 and lactate dehydrogenase at each time point. Table S4. Pearson’s correlation coefficient matrix of myomesin 3 and muscle tenderness at each time point. Table S5. Pearson’s correlation coefficient matrix of myomesin 3 and DOMS at each time point. Table S6. Pearson’s correlation coefficient matrix of myomesin 3 and ROM at each time point. [file 12576_2021_789_MOESM2_ESM.pdf]

Supplementary table 1. Pearson's correlation coefficient matrix of myomesin 3 and creatine kinase at each time point.

	CK 2h	CK 4h	CK 24h	CK 48h	CK 72h	CK 96h
MYOM3 2h	-0.488	-0.631	-0.17	-0.082	-0.299	-0.193
MYOM3 4h	-0.295	-0.519	-0.366	0.3	0.065	-0.017
MYOM3 24h	-0.422	-0.484	-0.177	-0.066	-0.173	-0.267
MYOM3 48h	0.292	-0.129	-0.45	0.955**	0.598	0.507
MYOM3 72h	0.35	-0.064	-0.428	0.966**	0.624	0.539
MYOM3 96h	0.308	-0.112	-0.444	0.960**	0.61	0.509

Myomesin 3 (MYOM3), creatine kinase (CK). All data are calculated as changes for the pre-exercise values. * $p<0.05$, ** $p<0.01$.

Supplementary table 2. Pearson's correlation coefficient matrix of myomesin 3 and myoglobin at each time point.

	Mb 2h	Mb 4h	Mb 24h	Mb 48h	Mb 72h	Mb 96h
**MYOM3 2h**	**-0.05**	**-0.121**	**-0.041**	**-0.015**	**-0.247**	**-0.053**
**MYOM3 4h**	**-0.019**	**-0.07**	**0.902****	**0.325**	**0.196**	**-0.194**
**MYOM3 24h**	**0.057**	**-0.013**	**0.933****	**-0.051**	**-0.062**	**-0.23**
**MYOM3 48h**	**-0.045**	**-0.024**	**0.193**	**0.976****	**0.727***	**0.139**
**MYOM3 72h**	**-0.049**	**-0.019**	**0.076**	**0.985****	**0.740***	**0.172**
**MYOM3 96h**	**-0.044**	**-0.021**	**0.179**	**0.980****	**0.737***	**0.147**

Myomesin 3 (MYOM3), myoglobin (Mb). All data are calculated as changes for the pre-exercise values. * $p<0.05$, ** $p<0.01$.

Supplementary table 3. Pearson's correlation coefficient matrix of myomesin 3 and lactate dehydrogenase at each time point.

	LDH 2h	LDH 4h	LDH 24h	LDH 48h	LDH 72h	LDH 96h
MYOM3 2h	-0.418	-0.593	-0.096	-0.379	-0.267	-0.287
MYOM3 4h	0.284	-0.165	0.494	0.117	0.141	0.017
MYOM3 24h	0.362	-0.07	0.542	-0.063	-0.164	-0.229
MYOM3 48h	0.094	-0.114	0.258	0.638	0.790*	0.585
MYOM3 72h	0.05	-0.104	0.193	0.65	0.814**	0.615
MYOM3 96h	0.088	-0.11	0.25	0.642	0.798**	0.588

Myomesin 3 (MYOM3), lactate dehydrogenase (LDH). All data are calculated as changes for the pre-exercise values. * $p<0.05$, ** $p<0.01$.

Supplementary table 4. Pearson's correlation coefficient matrix of myomesin 3 and muscle tenderness at each time point.

	TDN 24h	TDN 48h	TDN 72h	TDN 96h
MYOM3 24h	0.108	-0.119	-0.191	-0.203
MYOM3 48h	0.599	0.909**	0.777*	0.554
MYOM3 72h	0.584	0.925**	0.803*	0.582
MYOM3 96h	0.598	0.914**	0.784*	0.562

Myomesin 3 (MYOM3) and muscle tenderness (TDN). All data are calculated as changes for the pre-exercise values. * $p<0.05$, ** $p<0.01$.

Supplementary table 5. Pearson's correlation coefficient matrix of myomesin 3 and DOMS at each time point.

	VAS 24h	VAS 48h	VAS 72h	VAS 96h
MYOM3 24h	0.086	0.153	0.192	0.223
MYOM3 48h	0.106	0.736*	0.592	0.251
MYOM3 72h	0.09	0.720*	0.574	0.231
MYOM3 96h	0.099	0.740*	0.598	0.259

Myomesin 3 (MYOM3) and visual analogue scale (VAS) for delayed-onset muscle soreness (DOMS). All data are calculated as changes for the pre-exercise values. * $p<0.05$.

Supplementary table 6. Pearson's correlation coefficient matrix of myomesin 3 and ROM at each time point.

	ROM 24h	ROM 48h	ROM 72h	ROM 96h
MYOM3 24h	0.028	-0.114	-0.062	0.132
MYOM3 48h	0.618	0.169	0.042	0.507
MYOM3 72h	0.617	0.188	0.055	0.495
MYOM3 96h	0.623	0.174	0.045	0.506

Myomesin 3 (MYOM3) and ankle range of motion (ROM) in dorsal flexion. All data are calculated as changes for the pre-exercise values.
